# Peripherally Acting μ-Opioid Receptor Antagonists in the Management of Postoperative Ileus: a Clinical Review

**DOI:** 10.1007/s11605-020-04671-x

**Published:** 2020-08-10

**Authors:** Karim Chamie, Vishnukamal Golla, Andrew T. Lenis, Patrick M. Lec, Siamak Rahman, Eugene R. Viscusi

**Affiliations:** 1 Department of Urology, University of California Los Angeles Medical Center, Los Angeles, CA USA; 2 Department of Anesthesiology, University of California Los Angeles Medical Center, Los Angeles, CA USA; 3 Department of Anesthesiology, Sidney Kimmel Medical College at Thomas Jefferson University, 111 S 11th St, Ste G-8290, Philadelphia, PA 19107 USA

**Keywords:** Postoperative ileus, Opioid-induced constipation, Opioid analgesics, μ-Receptor antagonists, Alvimopan, Methylnaltrexone

## Abstract

Postoperative ileus (POI) and constipation are common secondary effects of opioids and carry significant clinical and economic impacts. μ-Opioid receptors mediate opioid analgesia in the central nervous system (CNS) and gastrointestinal-related effects in the periphery. Peripherally acting μ-opioid receptor antagonists (PAMORAs) block the peripheral effects of opioids in the gastrointestinal tract, while maintaining opioid analgesia in the CNS. While most are not approved for POI or postoperative opioid-induced constipation (OIC), PAMORAs have a potential role in these settings via their selective effects on the μ-opioid receptor. This review will discuss recent clinical trials evaluating the safety and efficacy of PAMORAs, with a focus on alvimopan (Entereg^®^) and methylnaltrexone (Relistor^®^) in patients with POI or postoperative OIC. We will characterize potential factors that may have impacted the efficacy observed in phase 3 trials and discuss future directions for the management and treatment of POI.

## Introduction

### Postoperative Ileus

Postoperative ileus (POI) is defined as a delay of normal gastrointestinal (GI) motility after surgery and can be secondary to surgical stress responses (i.e., bowel manipulation and/or resection), neurohormonal dysfunction, inflammation, fluid and electrolyte imbalances, and opioids (both endogenous and exogenous).^1^–^3^ Clinical features of POI include bloating, abdominal distention, nausea, vomiting, delay in oral intake, and pain.^4^ The incidence of POI has been reported in 10 to 30% of patients who have undergone colectomy, cholecystectomy, or other abdominal surgeries.^1^, ^5^ Multiple factors may affect the incidence of POI, including the type and duration of surgery,^6^, ^7^ inflammatory factors induced during surgery,^8^ and opioid dose^5^ and duration.^9^

POI is associated with decreased patient satisfaction, extended hospital length of stay, medical complications, increased hospital costs, subsequent surgical interventions, and higher readmission rates.^5^, ^10^, ^11^ Hospital costs in patients with POI are estimated to be double compared with those who have normal return of bowel function (median total hospital costs, $21,046 vs $10,945).^5^ As such, accelerating GI recovery after surgery is paramount in improving patient outcomes and decreasing costs.

Strategies to improve rates of POI have changed overtime. Traditional approaches to POI prevention include chewing gum, adequate fluid resuscitation, and administration of prokinetic drugs and laxatives.^3^ However, some of these measures are associated with additional risks or have limited or unproven efficacy.^3^ More contemporary multimodal strategies, termed enhanced recovery after surgery (ERAS) protocols, aim to limit the stress response to surgery.^3^ Examples of key interventions of the ERAS protocol are to encourage ambulation, early diet, and multimodal analgesia.^3^, ^12^–^15^ Peripheral μ-opioid receptor antagonists (PAMORAs) also play a critical role in many ERAS protocols, especially among patients undergoing open surgery.^3^ Herein, we review the role of μ-opioid receptors in GI motility, the use of PAMORAs after surgery to accelerate GI recovery, and discuss future directions.

### Peripherally Acting μ-Opioid Receptor Antagonists in GI Recovery

Opioid analgesics are often administered during and after surgical procedures for the management of intraoperative and postoperative pain, respectively.^16^ In addition to their intended central effect on pain receptors, opioids have an undesired influence peripherally in the GI tract, including decreased gastric motility and emptying, inhibited bowel propulsion, and altered fluid and electrolyte balance.^17^ Opioids profoundly affect the forward propulsive and coordinated peristaltic activity of the bowel leading to both POI and postoperative opioid-induced constipation (OIC).^16^, ^18^ Although OIC and POI are similar, POI involves the loss of forward propulsive motion of the GI tract after surgery.^19^

Opioid receptors, of which there are 3 main classes (μ, δ, κ), are ubiquitous in the central nervous system (CNS) and enteric nervous system.^17^ Nonselective μ-opioid receptor antagonists cross the blood-brain barrier and target both central and peripheral μ-opioid receptors.^20^, ^21^ For example, the nonselective μ-opioid receptor antagonist naloxone (Narcan^®^) has a long clinical history of successful use for the treatment of opioid overdose, reversing overdose-induced respiratory depression while reducing symptoms of acute withdrawal in opioid-dependent patients.^22^ Targeting of both central and peripheral μ-opioid receptors relieves constipation but also reverses opioid analgesia.^20^, ^21^

PAMORAs aim to mitigate the risk of POI while maintaining analgesia in patients receiving postoperative opioids by selectively inhibiting peripheral μ-opioid receptors.^23^ The effects of PAMORAs are limited to the periphery given their polarity, large structure, and low lipid solubility that prevents them from crossing the blood-brain barrier.^24^ As such, selective antagonism of the GI μ-opioid receptors blocks the peripheral effects of opioids, while maintaining the analgesic effects of opioids in the CNS.^23^, ^25^

Most of the approved PAMORAs are not indicated for POI or postoperative OIC, but they may have a role in the management of postoperative bowel dysfunction by virtue of their abovementioned mechanism of action.^19^ Currently, alvimopan and methylnaltrexone have the most significant data supporting their use for POI. Other PAMORAs, such as naloxegol, are less thoroughly studied with only small series demonstrating some efficacy for POI.^26^

## PAMORAs for POI

### Alvimopan (Entereg®)

Alvimopan is the only PAMORA indicated to accelerate the time to upper and lower GI recovery following partial bowel resection surgery with primary anastomosis (Table 1).^27^ It is limited to 15 total doses, which can only be administered in the hospital.^27^ Dosing guidelines recommend one 12-mg capsule 30 min to 5 h before surgery and one 12-mg capsule twice daily beginning the day after surgery until discharge for a maximum of 7 days.^27^ In clinical studies for the management of POI, alvimopan was administered orally (alvimopan 6 and/or 12 mg) preoperatively and continued following surgery.^28^–^33^

**Table 1:** Summary of alvimopan and methylnaltrexone use^27^, ^40^

Drug	Indications and usage	Route of administration	Dosing	Safety
Alvimopan (Entereg^®^)	Indicated to accelerate the time to upper and lower GI recovery following surgeries that include partial bowel resection with primary anastomosis REMS for short-term use	Oral	Preoperative and postoperative • 12 mg, 30 min to 5 h before surgery followed by 12 mg BID after surgery for up to 7 days In-hospital administration only • Maximum of 15 in-hospital doses	• Includes a boxed warning and REMS for short-term use because of the potential risk of MI with long-term use • Previous opioid exposure increases sensitivity • Not recommended in patients with severe hepatic impairment, end-stage renal disease, complete GI obstruction, surgical correction of complete bowel obstruction, pancreatic or gastric anastomosis
Methylnaltrexone (Relistor^®^)	Not indicated for OIC in the surgical setting or POI Indicated for treatment of OIC in adults with: • Chronic noncancer pain, including patients with chronic pain related to prior cancer or its treatment who do not require frequent opioid dosage escalation (injection and tablets) • Advanced illness or pain caused by active cancer who require opioid dosage escalation for palliative care (injection)	Oral or SC injection	For OIC in adult patients with chronic noncancer pain: • 450 mg per oral QD • 12 mg SC QD For OIC in adult patients with advanced illness: • One SC dose EOD, as needed according to weight	• Not recommended in patients with known or suspected GI obstruction and at an increased risk of recurrent obstruction • May cause severe or persistent diarrhea • May result in symptoms of opioid withdrawal

*BID* twice daily, *EOD* every other day, *GI* gastrointestinal, *MI* myocardial infarction, *OIC* opioid-induced constipation, *POI* postoperative ileus, *QD* once daily, *REMS* risk evaluation and mitigation strategy, *SC* subcutaneous

#### Alvimopan and GI Recovery

The safety and efficacy of alvimopan has been evaluated in patients undergoing major abdominal surgery, including bowel resection,^28^–^32^ hysterectomy,^30^, ^31^, ^33^ and radical cystectomy.^34^ The primary efficacy endpoint for the earlier alvimopan efficacy studies was time to recovery of GI function as demonstrated by the endpoint GI3.^29^, ^31^, ^32^ GI3 is a composite endpoint determined by the time that a patient first tolerated solid food (a marker for upper gastrointestinal function recovery) and the time that a patient first passed either flatus or had a bowel movement (a marker for lower gastrointestinal recovery).^29^–^32^ However, the GI3 endpoint did not demonstrate statistically significant differences compared with placebo in every alvimopan study (Table 2).^29^, ^31^, ^33^ Post hoc analysis of the data suggested that a secondary endpoint, GI2, was more objective. The GI2 endpoint is the same as the GI3 endpoint but eliminates flatus as a marker for lower GI recovery. Flatus has been found to be subjective and highly variable.^29^–^32^ As such, GI2 was preferentially utilized in subsequent alvimopan studies.^28^, ^29^, ^32^, ^33^ Indeed, across most studies, time to the GI2 endpoint, regardless of whether it was a primary or secondary endpoint, was reduced in the alvimopan group compared with that in the placebo group.^28^, ^29^, ^33^ Overall, alvimopan shortened the time to GI recovery and time to readiness for discharge (Table 2).^28^, ^30^, ^32^, ^35^

**Table 2:** Summary of efficacy data from alvimopan and methylnaltrexone pivotal clinical trials^28^, ^29^, ^31^–^33^, ^41^–^43^

Study	Study design	Type of surgery	Constipation type	Drug administration	Endpoint(s)	Treatments	Endpoint result
Alvimopan							
Wolff et al. 2004	Phase 3, randomized, double-blind, placebo-controlled	• Partial bowel resection with primary anastomosis • Radical hysterectomy	POI	• Oral • 2 h before surgery • After surgery, BID until discharge or for maximum of 7 days	Mean GI3, hour	• Placebo	120
						• Alvimopan 6 mg	105; *P* < 0.05
						• Alvimopan 12 mg	98; *P* < 0.001
					Mean GI2, hour	• Placebo	133
						• Alvimopan 6 mg	113; *P* = 0.013
						• Alvimopan 12 mg	105; *P* < 0.001
					Mean hospital discharge order written, hour	• Placebo	146
						• Alvimopan 6 mg	133; *P* = 0.070
						• Alvimopan 12 mg	126; *P =* 0.003
Delaney et al. 2005	Phase 3, randomized, double-blind, placebo-controlled	• Segmental colon resection • Simple or radical hysterectomy	POI	• Oral • 2 h before surgery • After surgery, BID until discharge or for maximum of 7 days	Mean GI3, hour	• Placebo	100.3
						• Alvimopan 6 mg	86.2; *P* = 0.003
						• Alvimopan 12 mg	92.8; *P* = 0.059
					Mean hospital discharge order written, hour	• Placebo	122
						• Alvimopan 6 mg	108; *P* < 0.001
						• Alvimopan 12 mg	115; *P* = 0.17
Viscusi et al. 2006	Phase 3, randomized, placebo-controlled, parallel group	• Bowel resection or total abdominal hysterectomy	POI	• Oral • 2 h before surgery • After surgery, BID for 7 days	Mean GI3, hour	• Placebo	224
						• Alvimopan 6 mg	220; *P* = 0.037*
						• Alvimopan 12 mg	221; *P* = 0.028*
Herzog et al. 2006	Phase 3, randomized, double-blind, placebo-controlled	• Simple hysterectomy	POI	• Oral • 2 h before surgery • After surgery, BID for 7 days	Mean GI3, hour	• Placebo	55.4
						• Alvimopan 12 mg	53.5; *P* = NS
					Mean GI2, hour	• Placebo	92.0
						• Alvimopan 12 mg	71.8; *P* < 0.001
					Mean hospital discharge order written, hour	• Placebo	68.6
						• Alvimopan 12 mg	66.3; *P* = NS
Buchler et al. 2008	Phase 3, randomized, double-blind, placebo- controlled	• Small or large bowel resection with primary anastomosis	POI	• Oral • 2 h before surgery • After surgery, BID until discharge or for a maximum of 7 days	Mean GI3 (SE), hour	• Placebo	92.6 (3.06)
						• Alvimopan 6 mg	84.2 (2.37); *P =* NS
						• Alvimopan 12 mg	87.8 (2.68); *P =* NS
					Mean GI2 (SE), hour	• Placebo	109.5 (3.41)
						• Alvimopan 6 mg	95.2 (2.40); *P* < 0.001
						• Alvimopan 12 mg	98.8 (2.71); *P =* 0.008
Ludwig et al. 2008	Phase 3b, randomized, double-blind, placebo-controlled	• Small or large bowel resection with primary anastomosis	POI	• Oral • 30 to 90 min before surgery • After surgery, BID until discharge or for up to a maximum of 7 days	Mean GI2, hour	• Placebo	112
						• Alvimopan 12 mg	92; *P* < 0.001
					Mean GI3, hour	• Placebo	98
						• Alvimopan 12 mg	82; *P* = NR
					Mean hospital discharge order written, hour	• Placebo	138
						• Alvimopan 12 mg	120; *P* < 0.001
Methylnaltrexone							
Anissian et al. 2012	Phase 2, randomized, double-blind, placebo-controlled	• Orthopedic surgery	Acute-onset OIC	• SC • QD for up to 4 or 7 days	Median time to first bowel movement, hour	• Placebo	50.9
						• Methylnaltrexone 12 mg	15.8; *P* = 0.02
Viscusi et al. 2013	Phase 2, randomized, double-blind, placebo-controlled	• Segmental colectomy	POI	• IV • Within 90 min after surgery • Every 6 h thereafter until patient tolerates solid food for 24 h, is discharged, or for a maximum of 7 days	Mean time to first bowel movement (SD), hour	• Placebo	118.1 (10.3)
						• Methylnaltrexone 0.30 mg/kg	98.0 (5.7); *P* = 0.038
					Mean discharge eligibility (SD), hour	• Placebo	148.7 (17.2)
						• Methylnaltrexone 0.30 mg/kg	116.1 (6.9); *P* = 0.049
Yu et al. 2011	Phase 3, randomized, double-blind, placebo-controlled (2 studies)	• Segmental colectomy	POI	• IV • Within 90 min after surgery • Every 6 h thereafter until 24 h after the return of bowel function, discharge, or for a maximum of 10 days	Median time to first bowel movement, days: Study 1	• Placebo	4.23
						• Methylnaltrexone 12 mg	4.14; *P* = NS
						• Methylnaltrexone 24 mg	3.88; *P* = NS
					Median time to first bowel movement, days: Study 2	• Placebo	4.52
						• Methylnaltrexone 12 mg	4.61; *P* = NS
						• Methylnaltrexone 24 mg	4.36; *P* = NS

*BID* twice daily, *IV* intravenous, *NR* not reported, *NS* not significant, *OIC* opioid-induced constipation, POI postoperative ileus, *QD* once daily, *SD* standard deviation, *SE* standard error, *SC* subcutaneous

*After adjustment for multiple comparisons

#### Alvimopan and Hospitalization

The effect of alvimopan has also been studied in large databases in order to evaluate the effects on length of stay and postoperative complications. For example, the Premier Perspective database is an observational propensity-matched cohort study that includes hospitalized postsurgical patients from a network of more than 400 hospitals.^36^ Using this database, Steele and colleagues evaluated the effect of alvimopan on clinical outcomes and healthcare utilization in patients undergoing bowel resection.^36^ Compared with matched cohorts (*n* = 18,559), patients receiving alvimopan (*n* = 18,559) had significantly reduced postoperative length of stay (mean ± standard deviation, 4.62 ± 2.45 days vs 5.24 ± 3.35 days; *P* < 0.001).^36^ In addition, alvimopan was associated with significantly reduced postoperative GI complications, urinary tract infections, and postoperative infections (all *P* < 0.001).^36^ Cardiovascular, pulmonary, and thromboembolic events were also significantly reduced in the alvimopan group when compared with those in matched controls (*P* < 0.001).^36^ The authors noted that these improved outcomes may have been a result of shorter hospital length of stays and the implementation of ERAS protocols.^36^

In a recent subanalysis of patients who underwent bowel resection from the Premier Perspective database, alvimopan (*n* = 15,719) reduced the incidence of POI (*P* < 0.001), POI-related readmission (*P* < 0.01), and length of hospital stay (*P* < 0.001) compared with matched controls (*n* = 37,229).^37^ Cardiovascular complications and in-hospital mortality were reduced in patients at risk for POI who received alvimopan compared with matched controls.^37^ Similarly, in a study of patients undergoing radical cystectomy, the prolonged length of stay (> 7 days) was significantly lower for patients receiving alvimopan compared with that for those receiving placebo (32.9% vs 51.5%; *P* < 0.01).^34^

Conflicting data, however, have been reported regarding the utility of alvimopan in patients undergoing surgery with intestinal anastomosis. In a recent small retrospective analysis of alvimopan (*n* = 55) and matched controls (*n* = 58), the authors found no significant difference in length of hospital stay (4.6 vs 4.8 days; *P* = 0.72) or mean time to return to bowel function (68.5 vs 67.3 h; *P* = 0.83).^38^ This study demonstrated that patients treated with alvimopan incurred significantly greater charges for medical and surgical supplies, pathology and cytology services, operating room charges, and therapy charges (*P* < 0.05).^38^ However, these increases in costs were largely associated with surgical time and a shift in hospital-wide accelerated recovery efforts; they may also be due to differences in surgical complexity between groups.^38^ Nevertheless, this suggests that patient selection is important in deciding who should receive ERAS interventions, including PAMORAs, such as alvimopan.

#### Alvimopan Safety

In the majority of studies evaluating the safety and efficacy of alvimopan, the exclusion criteria prohibited preoperative use of opioids.^28^–^31^, ^33^ As such, alvimopan is contraindicated in patients who are taking therapeutic doses of opioids for greater than 7 days before starting alvimopan.^27^ Further, the alvimopan label warns that recent exposure to opioids may increase the risk of abdominal pain, nausea, vomiting, and diarrhea since patients recently exposed to opioids may be more sensitive to the effects of PAMORAs.^27^

The duration of treatment with alvimopan is limited to short-term use in the hospital (15 doses) as noted in the boxed warning within the label.^27^ As such, an FDA risk evaluation and mitigation strategy is required to ensure that alvimopan use is restricted to the hospital setting.^27^ Secondly, the box warning also states that the incidence of MI was greater in patients receiving alvimopan compared with that in those receiving placebo in a 12-month study of patients treated with opioids for chronic noncancer pain.^27^ The increased incidence of MI and cardiovascular events with long-term treatment was not significantly different between treatment groups, but there was a numeric difference noted between groups.^39^ MI and cardiovascular events were suffered by 7 (1.3%) and 14 (2.6%) patients who received alvimopan, respectively, compared with 0 (0%) and 3 (1.1%) patients who received placebo, respectively. All events occurred in patients who were at a high risk for or had established cardiovascular disease.^39^ While this was originally thought to be an overall function of the drug class, subsequent phase 3 and dedicated long-term safety studies of alvimopan in POI have failed to identify any increased risk for cardiovascular adverse events.^36^, ^37^ Importantly, unlike the increased cardiovascular risks observed in the 12-month study, there was no increased risk of MI observed in short-term trials (up to 7 days) with alvimopan.^27^

### Methylnaltrexone (Relistor®)

Methylnaltrexone (formulated as a subcutaneous [SC] and oral dosage) is a PAMORA indicated for the treatment of OIC in adults with chronic noncancer pain, including patients with chronic pain related to prior cancer who are on a stable opioid dose.^40^ SC methylnaltrexone is also indicated for the treatment of OIC in adults with advanced illness or active cancer who require an increase in opioid dose for palliative care (Table 1).^40^

For OIC in patients with chronic noncancer pain, methylnaltrexone is given as an SC injection of 12 mg SC once daily or an oral 450-mg dose in the morning. For patients with advanced illness, the SC methylnaltrexone dosage is based on body weight and administered once every other day as needed.^40^ Patients who weigh 38 to < 62 kg should receive an 8-mg dose, and patients who weigh 62 to 114 kg should receive a 12-mg dose. Those who fall outside this weight range should receive a dose of 0.15 mg/kg.^40^

While neither the injection nor the oral formulation of methylnaltrexone is indicated for POI or postoperative OIC,^40^ trials have been conducted in the postsurgical setting for both potential indications.^41^–^43^ These studies and their results are summarized in Table 2 and discussed below.

#### Methylnaltrexone Efficacy and Safety

A double-blind, randomized, parallel-group, placebo-controlled phase 2 study was performed to assess the safety and efficacy of SC methylnaltrexone in patients with acute-onset OIC after orthopedic surgery.^41^ In this small study, patients received once-daily medication, either methylnaltrexone 12 mg (*n* = 18) or placebo (*n* = 15), for up to 4 or 7 days.^41^ At 2 and 4 h postinjection, a significantly higher percentage of methylnaltrexone-treated patients achieved laxation (bowel movement) versus placebo (2 h, 33.3% vs 0%, *P* = 0.021; 4 h, 38.9% vs 6.7%, *P* = 0.046) (Table 2).^41^ Time to response was also significantly reduced with SC methylnaltrexone treatment versus placebo (median 15.8 h vs 50.9 h, *P* = 0.02).^41^ In addition, the rates of adverse events (AEs) were similar between the 2 groups, with 33.3% and 26.7% of patients in the SC methylnaltrexone and placebo groups, respectively, reporting at least 1 treatment-emergent AE.^41^ Most AEs reported were GI-related and considered to be possibly related to the study medication.^41^ No cardiac AEs were reported and no safety signals or pattern of concern was visualized by ECG,^41^ suggesting that methylnaltrexone exposure does not share the same cardiac risks and precautions as alvimopan. These results demonstrated that SC methylnaltrexone was effective in improving GI recovery and was generally well-tolerated in patients with acute-onset OIC.^41^

Another phase 2, double-blind, randomized, controlled trial conducted in adults with POI after undergoing segmental colectomy showed that intravenous (IV) methylnaltrexone (0.30 mg/kg in 50 mL of saline; *n* = 33), compared with placebo (*n* = 32), significantly decreased the time to first bowel movement.^42^ The study medication was administered within 90 min following the surgical procedure and was repeated every 6 h until solid food was tolerated for 24 h; the patient was discharged, or the patient had received treatment for a maximum of 7 days.^42^ Compared with placebo, IV methylnaltrexone significantly accelerated the mean time to first bowel movement by 20 h (98.0 vs 118.1 h; *P* = 0.038) and reduced the mean time to discharge eligibility by 33 h (116.1 vs 148.7 h; *P* = 0.049).^42^ In this study, methylnaltrexone was generally well-tolerated. In fact, GI AEs, including nausea, vomiting, and abdominal pain, were more frequently reported in the placebo group compared with that in the IV methylnaltrexone group.^42^ Notably, the incidence of POI was reduced 2-fold in IV methylnaltrexone–treated patients compared with that in those receiving placebo (6% vs 16%).^42^

In a recent retrospective analysis of patients who underwent robotic-assisted radical cystectomy for bladder cancer, length of hospital stay, time to flatus and bowel movement, a composite of GI symptoms, episodes of severe pain, and daily opioid utilization were evaluated comparing SC methylnaltrexone (8 mg in patients < 65 kg and 12 mg in patients ≥ 65 kg; *n* = 29) with controls (*n* = 29).^44^ The median length of hospital stay was significantly reduced with methylnaltrexone treatment compared with the control group (4 vs 7 days, *P* < 0.01), which contributed to a significant reduction in the total cost of hospital stay by more than $10,500 USD per patient. The use of methylnaltrexone did not impact daily opioid usage or daily pain scores. In fact, there was a reduction in the number of severe pain episodes observed in methylnaltrexone-treated patients. Although there were no significant differences in the median time to flatus or bowel movement between patients who received methylnaltrexone compared with those patients in the control group, patients receiving methylnaltrexone had significantly fewer GI complications (methylnaltrexone cohort [10.3%] vs no methylnaltrexone cohort [44.8%], *P* < 0.01). The authors suggested that this finding may imply that the reduction of GI symptoms (bloating, distention, or discomfort) may be a factor in accelerated patient discharge.

#### Potential Factors Contributing to Lack of Efficacy of Methylnaltrexone in the Treatment of POI in Phase 3 Trials

The efficacy demonstrated in phase 2 trials could not be replicated in larger phase 3 trials. These phase 3 efficacy results were unexpected given the promising data from several phase 2 trials, but multiple factors may have contributed to the perceived lack of efficacy, some of which are reviewed here (Table 3).

**Table 3:** Potential factors contributing to lack of efficacy of methylnaltrexone in the treatment of POI in phase 3 trials^4^, ^41^, ^43^, ^46^

Total amount of opioid administered to patients is not reported and may not have been sufficient to activate μ-opioid receptors	
Multimodal treatment approaches and postoperative care may not have been matched between treatment groups	
Endpoint selection may factor into observed lack of efficacy	
Prior use of opioids may have saturated μ-opioid receptors, leading to ineffective methylnaltrexone treatment	
Dosing was inconsistent across studies; IV methylnaltrexone may not be the best route of administration for POI	
Genetic polymorphisms and concomitant drug usage may produce interpatient variability	

The total amount of opioid administered to patients in the methylnaltrexone studies has not been reported.^4^ This is important as PAMORAs function to reduce POI by directly inhibiting opioid-mediated activation of μ-opioid receptors in the GI tract.^4^ In the study inclusion criteria, patients using opioids prior to surgery were ineligible to participate in many of the alvimopan studies whereas these patients were not excluded from the methylnaltrexone studies.^28^–^31^, ^33^ Ideally, treatment with a μ-opioid receptor antagonist would start preoperatively in an opioid-naive patient, beginning prior to the exposure of any opioids during anesthesia.^4^ In the alvimopan studies, preoperative dosing was used instead of the postoperative dosing used in the methylnaltrexone POI studies. This is critical as high doses of opioids are generally given intraoperatively and may have saturated μ-opioid receptors prior to treatment with methylnaltrexone.^4^ Furthermore, the doses used in the methylnaltrexone POI studies were the same as those used in the OIC studies. This is in contrast to the alvimopan analyses that used doses considerably higher in the POI studies compared with the OIC studies.^32^, ^45^

Endpoint selection may also have contributed to variable efficacy results. In contrast to the composite endpoints used in the alvimopan trials (GI2 or GI3), single endpoints (i.e., time to first bowel movement or flatus) were assessed in the methylnaltrexone studies.^4^ Flatus, for example, when included in the GI3 composite endpoint, was found in the alvimopan studies to be a poor marker for GI recovery.^29^

Additionally, the phase 3 studies of methylnaltrexone utilized IV delivery, which introduced different dosing parameters than those used in the efficacious phase 2 OIC study.^41^, ^43^ Potential differences in the pharmacokinetics and pharmacodynamics between IV and SC dosing could have affected the efficacy of the drug.^4^ Finally, some opioids are P-glycoprotein substrates, requiring a functional P-glycoprotein for analgesic effect.^46^ Genetic polymorphisms and concomitant drug usage may produce interpatient variability in the expression of P-glycoprotein or other drug efflux transporters.^46^ This, in turn, may affect the absorption, distribution, or efficacy of PAMORAs.^46^

#### Long-term Use and Effects on Survival in Methylnaltrexone Studies for OIC

Although data on the use of methylnaltrexone in POI or postoperative OIC is limited, studies have shown safety and efficacy in trials up to 48 weeks in patients with noncancer pain^47^ and improved survival in cancer patients^48^ when used for OIC.

Long-term use of methylnaltrexone in patients with noncancer pain and OIC who received SC methylnaltrexone 12 mg daily for 48 weeks was assessed in a phase 3 open-label trial.^47^ A significant increase in mean weekly bowel movement rate change from baseline (*P* < 0.001) was observed through the entire 48-week period.^47^ Similar to other studies of shorter duration, GI-related AEs were the most commonly reported AEs (i.e., abdominal pain 24.0%, diarrhea 16.4%, nausea 15.1% of patients). These rates are consistent with a long-term open-label study that found that GI-related AEs ranged from 15.5 to 19.3%.^47^ This study demonstrated that SC methylnaltrexone provided consistent long-term treatment for OIC without new safety concerns, suggesting that methylnaltrexone is safe and effective to use in the long term.^47^

A retrospective post hoc analysis of data pooled from 2 independent randomized, placebo-controlled clinical trials of SC methylnaltrexone in patients with advanced terminal cancer and OIC assessed whether treatment could influence survival.^48^ This analysis demonstrated that SC methylnaltrexone significantly increased the median overall survival compared with placebo (76 days, 95% confidence interval [CI] 43–109, vs 56 days, 95% CI 43–69; *P* = 0.033), indicating that methylnaltrexone may have an impact beyond relief of constipation, including a direct effect of methylnaltrexone on cellular targets related to the morphine receptor.^48^ Morphine antagonists have been shown to reduce tumor growth in several cancer in vivo models.^49^–^52^

### Other PAMORAs

Naloxegol and naldemedine are two other PAMORAs indicated for the treatment of OIC in patients with chronic noncancer pain, but do not have an indication for POI.^53^, ^54^ Although no studies have been conducted in POI treatment, a small study has been published in POI prevention. In an active comparator analysis, perioperative administration of naloxegol was compared with alvimopan among adult patients with bladder cancer who underwent a radical cystectomy.^26^ Among 130 patients, no differences were observed between those receiving naloxegol or alvimopan with respect to the development of POI or hospital length of stay. The authors postulated that although no other studies have been conducted with naloxegol, this active comparator study may help support the use of naloxegol as a less expensive alternative to alvimopan. To date, data evaluating the use of naldemedine for OIC in the surgical setting or for the treatment or prevention of POI have not been published.

## Future Direction

Presently, with the exception of the small naloxegol versus alvimopan analysis,^26^ direct comparisons between PAMORAs are not possible because comparator studies between these agents have not been performed. Head-to-head studies are needed to make definitive conclusions as protocols and endpoints differ among the various studies.

The pathophysiology of POI is multifactorial and not exclusively related to opioid ligands. Therefore, a multimodal treatment strategy that takes into account inhibition of μ-opioid receptors with PAMORAs, while including a variety of techniques or interventions with other mechanisms of action, may be needed.^4^ It is likely that the use of PAMORAs concurrently with these adjunct therapies will result in clinically meaningful acceleration of GI recovery after surgery^4^ beyond what can be achieved by any single intervention alone.

Differences in surgical approach (e.g., open, robot-assisted, or laparoscopic) and types of procedures (e.g., cystectomy vs colectomy) between studies may cause differences in rates of postoperative OIC and POI and the observed efficacy of PAMORAs.^1^, ^5^–^7^ Furthermore, the alvimopan doses studied were those specific for use in POI,^28^–^33^ whereas in the postsurgical setting, the doses of methylnaltrexone studied were often those used in OIC studies.^41^, ^43^ As such, further analysis into the appropriate dose of methylnaltrexone for POI may be necessary.

## Conclusions

Pharmacologic options for the treatment or prevention of POI are limited. Currently, alvimopan is the only PAMORA approved for POI that improves GI recovery after surgery and reduces the length of hospital of stay. Although methylnaltrexone is not indicated for POI, studies have shown that it effectively reverses unwanted opioid-induced GI side effects while preserving CNS-mediated analgesia in specific surgical settings. Further studies with methylnaltrexone are warranted to determine its utility in prevention and/or treatment of POI. Given the inconsistencies observed between current studies, future trial designs should be carefully considered in order to evaluate efficacy.
